# Neural Decoding and “Inner” Psychophysics: A Distance-to-Bound Approach for Linking Mind, Brain, and Behavior

**DOI:** 10.3389/fnins.2016.00190

**Published:** 2016-04-28

**Authors:** J. Brendan Ritchie, Thomas A. Carlson

**Affiliations:** ^1^Laboratory of Biological Psychology, Brain and Cognition Unit, KU LeuvenLeuven, Belgium; ^2^Department of Philosophy, University of MarylandCollege Park, MD, USA; ^3^Perception in Action Research Centre, Department of Cognitive Science, Macquarie UniversitySydney, NSW, Australia; ^4^ARC Centre of Excellence in Cognition and its Disorders, Macquarie UniversitySydney, NSW, Australia

**Keywords:** inner psychophysics, MVPA, signal detection theory, visual object categorization, reaction times

## Abstract

A fundamental challenge for cognitive neuroscience is characterizing how the primitives of psychological theory are neurally implemented. Attempts to meet this challenge are a manifestation of what Fechner called “inner” psychophysics: the theory of the precise mapping between mental quantities and the brain. In his own time, inner psychophysics remained an unrealized ambition for Fechner. We suggest that, today, multivariate pattern analysis (MVPA), or neural “decoding,” methods provide a promising starting point for developing an inner psychophysics. A cornerstone of these methods are simple linear classifiers applied to neural activity in high-dimensional activation spaces. We describe an approach to inner psychophysics based on the shared architecture of linear classifiers and observers under decision boundary models such as signal detection theory. Under this approach, distance from a decision boundary through activation space, as estimated by linear classifiers, can be used to predict reaction time in accordance with signal detection theory, and distance-to-bound models of reaction time. Our “neural distance-to-bound” approach is potentially quite general, and simple to implement. Furthermore, our recent work on visual object recognition suggests it is empirically viable. We believe the approach constitutes an important step along the path to an inner psychophysics that links mind, brain, and behavior.

## 1. Mapping a route from mind to brain: the dream of an inner psychophysics

A fundamental challenge for cognitive neuroscience is to explain how the primitives of psychological theory are neurally implemented (Davis and Poldrack, 2013). Theories and models aimed at meeting this challenge are the modern manifestation of what Fechner (1860/1966) called “inner” psychophysics: the theory of the precise mapping between mental quantities and the brain. In Fechner's own time, inner psychophysics remained a dream (Scheerer, 1992). Even today, concrete proposals remain elusive. Indeed, many have wondered whether cognitive neuroscience is even up to the challenge (Price and Friston, 2005; Coltheart, 2006; Feldman Barrett, 2009; Poldrack, 2010). We side with those who have argued, more optimistically, that the field requires a shift in thinking for progress to continue (de Wit et al., 2016).

The key to inner psychophysics, we believe, is using psychological models, applied to neural activity, to predict behavior (Werner and Mountcastle, 1963; Britten et al., 1992). Consider the traditional motivation for using behavioral measures in (“outer”) psychophysics. Since the mind cannot be measured directly, Fechner and others reasoned that behavior can serve as a proxy to estimate stimulus-driven variation in mental quantities and processes. Similar reasoning supports behavioral measures as the key to developing “linking” hypotheses from psychological theory to the brain (Brindley, 1960; Teller, 1984). If a neural component implements a primitive identified by some theory or model, then we should be able to predict behavioral variation from its functional organization (Forstmann et al., 2011). We focus on *representational* linking hypotheses: how are the representations posited by psychological theory implemented by the brain in a manner that predicts behavior?

We propose that multi-variate pattern analysis (MVPA), or neural “decoding,” methods provide one starting point for the development of an inner psychophysics, and representational linking hypotheses. These methods have allowed researchers to investigate the information latent in neural activity patterns, and uncover the structure and content of the brain's population code (Kriegeskorte and Kievit, 2013; Haxby et al., 2014; Haynes, 2015). A cornerstone of these methods are linear classifiers applied to high-dimensional neural *activation spaces*. Here we present a simple approach for developing representational linking hypotheses based on the shared architecture of linear classifiers and observers under decision boundary models such as signal detection theory (Green and Swets, 1966). We also review our work on visual object categorization that lends empirical support to the approach (Carlson et al., 2014; Ritchie et al., 2015), and connect the approach to research on the neural loci of decision-making (Gold and Shadlen, 2007).

## 2. What can decoding contribute to inner psychophysics? biological vs. psychological plausibility

The suitability of MVPA methods for investigating neural representation, and developing representational linking hypotheses, can be motivated in part by their biological and (potential) psychological plausibility.

A common assumption in cognitive neuroscience is that the brain utilizes “population codes”: internal representations are implemented in distributed patterns of neural activity—incidentally, an idea somewhat anticipated by Fechner's (1882/1987) discussion of memory. If the brain uses population codes it may face a multivariate classification problem when differentiating these neural patterns. If this differentiation is achieved by a linear combinations of inputs, then we should be able to decode the contents of the encoding patterns of activity using classifiers that mirror the linear operations the brain employs. In decoding analyses, activation spaces are reconstructed from patterns of neural activity, and a linear classifier is trained to discriminate between the patterns for different experimental conditions. If the classifier performs significantly above chance, then minimally it can be inferred that information about the conditions is latent, and accessible, from the patterns of neural activity (Kriegeskorte and Bandettini, 2007). The biological plausibility of the linear classifiers also suggests that the information may be explicitly represented by the patterns.

While MVPA offers one starting point for developing an inner psychophysics, the biological plausibility of linear classifiers does not alone establish a connection between activation spaces and observer psychology. As de Wit et al. (2016) emphasize, that a classifier can learn to discriminate patterns of neural activity shows that information is latent, and perhaps represented, but not necessarily how it is being used, or is usable, by the observer (Cox and Savoy, 2003; Williams et al., 2007). In other words, the biological plausibility of linear classifiers is not enough to show that they are *psychologically* plausible, which also requires linking a psychological theory to an activation space. Fortunately, there is a long tradition in psychology of modeling the structure of psychological spaces to predict behavior (Attneave, 1950; Shepard, 1964; for a more recent perspective, see: Gärdenfors, 2000). All quantitative models of categorization within this tradition hold that tokens of a representation occupy different points in a space, and how these points are positioned in the space, based on some similarity or distance function, drives mental processes and behavior (Ashby and Maddox, 1993). For example, in prototype models (e.g., Posner and Keele, 1968) discriminability of a stimulus is determined by the distance of a representation to the central tendency of a category distribution in the space, and in exemplar models (e.g., Nosofsky, 1986) discriminability is determined by the relative similarity of the representation to all other exemplar representations in the space. The high-dimensional activation spaces reconstructed using MVPA may conform to similar principles of organization identified in these quantitative models of psychological space (Op de Beeck et al., 2008; Davis and Poldrack, 2013; Kriegeskorte and Kievit, 2013; Haxby et al., 2014). In which case, representational linking hypotheses can be developed by applying principles from models of psychological space to activation spaces.

One straightforward approach is to directly compare the structure of a psychological space to an activation space. Several studies using fMRI (Edelman et al., 1998; Mur et al., 2013; Charest et al., 2014; Sha et al., 2015; Bracci and Op de Beeck, 2016), cellular recordings (Op de Beeck et al., 2001) and MEG (Wardle et al., 2016), have constructed psychological spaces for stimuli from judgments of visual similarity, and compared them to activation spaces constructed using methods such as representational similarity analysis (RSA), which estimates the pair-wise (dis)similarity between patterns of neural activity for different conditions (Kriegeskorte et al., 2008a). A robust correlation between the two spaces suggests the activation spaces might implement the representations that are driving the similarity judgments. This similarity-based approach reflects the psychological plausibility of methods like RSA. Although seldom noticed in cognitive neuroscience, linear classifiers are also psychologically plausible, as we will illustrate.

## 3. A step in the right direction: a psychologically plausible neural distance-to-bound approach

As used in MVPA, linear classifiers specify a decision boundary through an activation space in order to discriminate between neural patterns produced by different experimental conditions. In general form this decision process is equivalent to that of the human observer as posited by (linear) decision boundary models of categorization for multivariate stimuli (Ashby and Gott, 1988; Ashby and Maddox, 1990). To illustrate the close correspondence of decoding methods with these models, consider that Naïve Bayes classifiers, applied after linear discriminate analysis (LDA), and observers under signal detection theory (SDT) share a common organization. More specifically, they make the same assumptions concerning: (i) distributions of evidence/data, and (ii) the evaluation of evidence/data by the classifier/observer.

**Figure 1:**
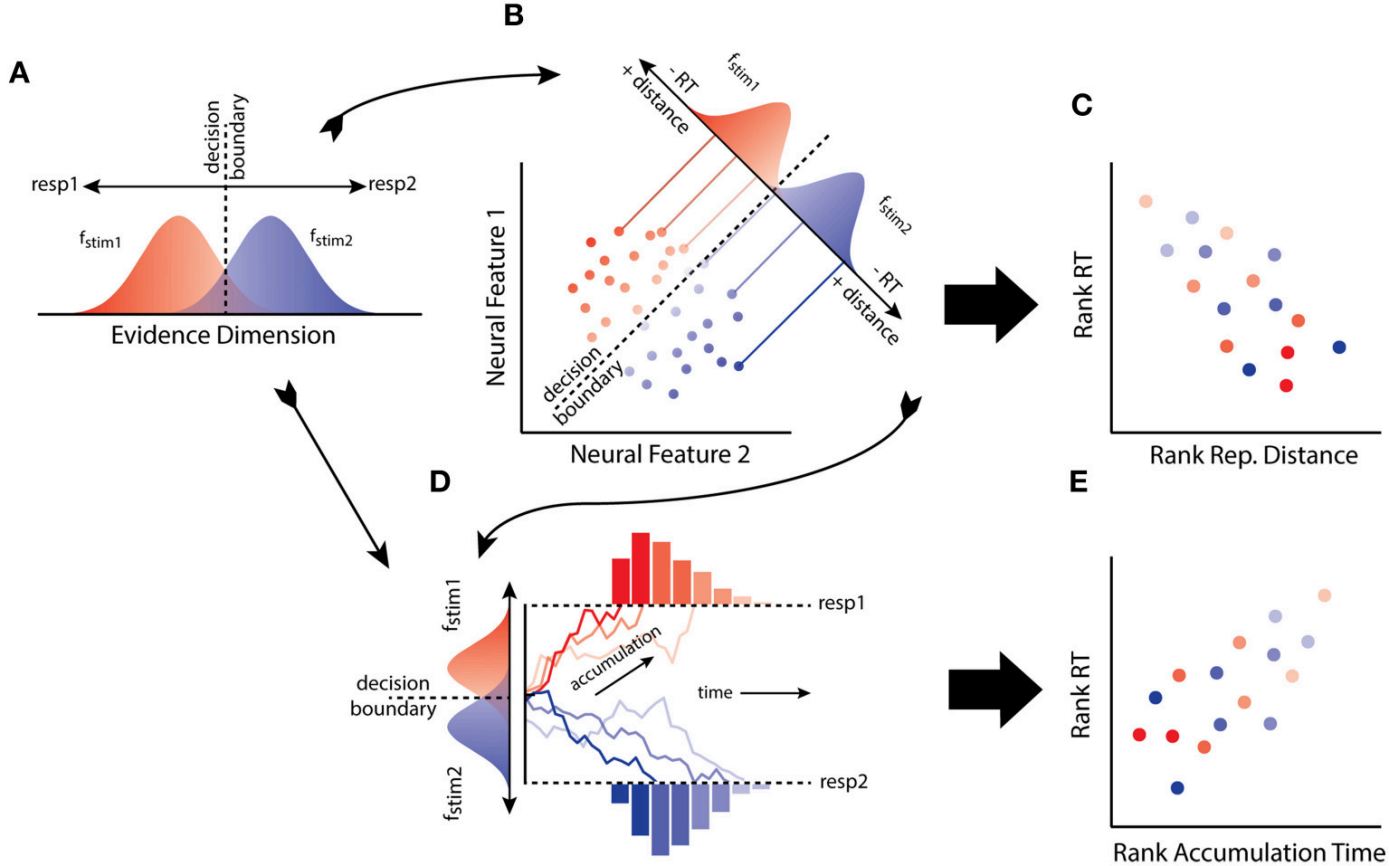
**(A)** The model of the observer from signal detection theory (SDT) maps to **(B)** the distributions of patterns of neural activity as reconstructed using neural decoding. **(C)** From this connection it is predicted that RT will negatively correlate with distance from a decision boundary through activation space. **(D)** The model of the observer from SDT, and the distributed patterns of neural activity, can also be linked to the evidence accumulation process. **(E)** Thus another prediction from the hypothesis is that distances from a decision boundary in activation space reflects the mean accumulation rate, and hence RT should correlate with accumulation rates determined by representational distance.

At an abstract level, SDT specifies a number of primitives that mediate the relationship between stimulus and behavior (Figure 1A). Consider a simple task in which an observer must discriminate and map two stimuli (*stim1, stim2*) to two responses (*resp1, resp2*). The input produced by a stimulus is characterized as a sample from one of two distributions (*f*_*stim*1_, *f*_*stim*2_) along an evidence dimension, e.g., brightness, with response choice resulting solely from a rule applied to a decision variable (assuming equal stimulus probabilities and outcome utilities). Traditionally, the decision variable was the value of the log-likelihood ratio of the evidence, given the available hypotheses (i.e., the logarithm of the ratio of the height of *f*_*stim*1_ and *f*_*stim*2_ at a point on the evidence dimension). Assuming no response bias, the decision rule states that the observer selects the response with greater value in the ratio, resulting in a decision boundary along the evidence dimension. Under the usual distributional assumptions of normalcy and equal variance, the measure of observer sensitivity generated by the model, *d'*, is the difference (or distance) between the means of *f*_*stim*1_ and *f*_*stim*2_ (it is also closely related to Fechner's own measure of sensitivity; Link, 1994). Architecturally, this model requires an internal stimulus representation along with a decision process that determines choice behavior given the information made explicit by the representation.

LDA is a technique for transforming a space to maximize between class variance (Duda et al., 2001). In the simplest case, a 2D-space is replaced with a single discriminant axis onto which each data point is projected. Importantly, it is assumed that the distributions for the classes along each dimension (or “feature”) are normal and of equal variance. If we further assume the dimensions are independent, then we have a Naïve Bayes classifier, which guesses based on the summed ratios of the posterior probabilities for each class along each dimension. If we take the logarithm of the ratio, and assume equal prior probabilities, then the classifier uses a decision rule applied to the log-likelihood ratio (Pereira et al., 2009). When a 2D space is projected to a single discriminant axis, the architectures of the classifier and the SDT observer are identical. In the multi-dimensional case, the classifier is akin to the decision boundary observer under the multi-dimensional generalization of SDT, when dimensions are independent (Ashby and Townsend, 1986).

An initial implication of this equivalence is that one may be able, in principle, to achieve close correspondence between classifier and observer performance. For example, consider the results of Philiastides and Sajda (2006) who observed similar psychometric and “neurometric” functions for human and classifier performance, when using an LDA classifier applied to EEG data. Not only do their results take on a new theoretical significance in light of the above equivalence, but methodologically their application of a sensitivity measure to classifier performance seems even more appropriate since the measure presupposes the very architecture that the classifier possesses (Tanner, 1956).

A further implication of the equivalence relates to reaction time (RT) and the speed of transforming representations of a stimulus into a decision. A simple feature of perceptual decision-making, as first characterized by SDT, is that the quality of evidence for an observer varies in its uncertainty (Tanner and Swets, 1954). In particular, evidence close to the observers decision boundary, or criterion, is more ambiguous, reflecting the greater likelihood of the evidence under the alternative hypotheses. In contrast, evidence far from the boundary is less ambiguous, reflecting greater likelihood of the evidence under one of the hypotheses about the source of the stimulus. Thus, relative to some decision boundary, evidence quality tends to vary with distance. RT also tends to vary with the quality of evidence: lower quality evidence results in longer decision times compared to high-quality evidence. A simple consequence of this familiar picture from decision boundary models (e.g., SDT), as developed with distance-to-bound models of choice and RT, is that distance from a decision boundary will negatively correlate with RT (Pike, 1973; Ashby and Maddox, 1994).

LDA classifiers learn to discriminate between activity patterns by positioning a decision boundary along a discriminant axis. If an activation space provides the evidence being utilized by an observer (Figure 1B), then one possibility is that distance from a classifier boundary will predict RT (Figure 1C). Such a result would suggest that an activation space implements an explicit representation of stimulus information that is used by the observer in a psychologically plausible manner. When decision boundary models like SDT were first developed, it was presumed that the evidence utilized by an observer was some unknown state of neural activity (Swets et al., 1961; Werner and Mountcastle, 1963). The neural distance-to-bound approach we have described provides a means of making good on this presumption. The approach is potentially quite general and is simple to implement as it relies on familiar MVPA and behavioral methods. We have also conducted experiments to test the approach.

### 3.1. Neural distance-to-bound predicts reaction time for object categorization

Two of our recent experiments on visual object categorization provide tangible evidence in support of neural distance-to-bound as a viable approach to inner psychophysics (Carlson et al., 2014; Ritchie et al., 2015). In both experiments, subjects were tasked with judging as quickly and accurately as possible whether object exemplars were animate or inanimate (i.e., “capable of self-movement”). RT for the task was then related to activation spaces reconstructed using fMRI and MEG decoding. Our prediction was that RT would negatively correlate with representational distances from a decision boundary computed using LDA (Figure 1C).

**Figure 2:**
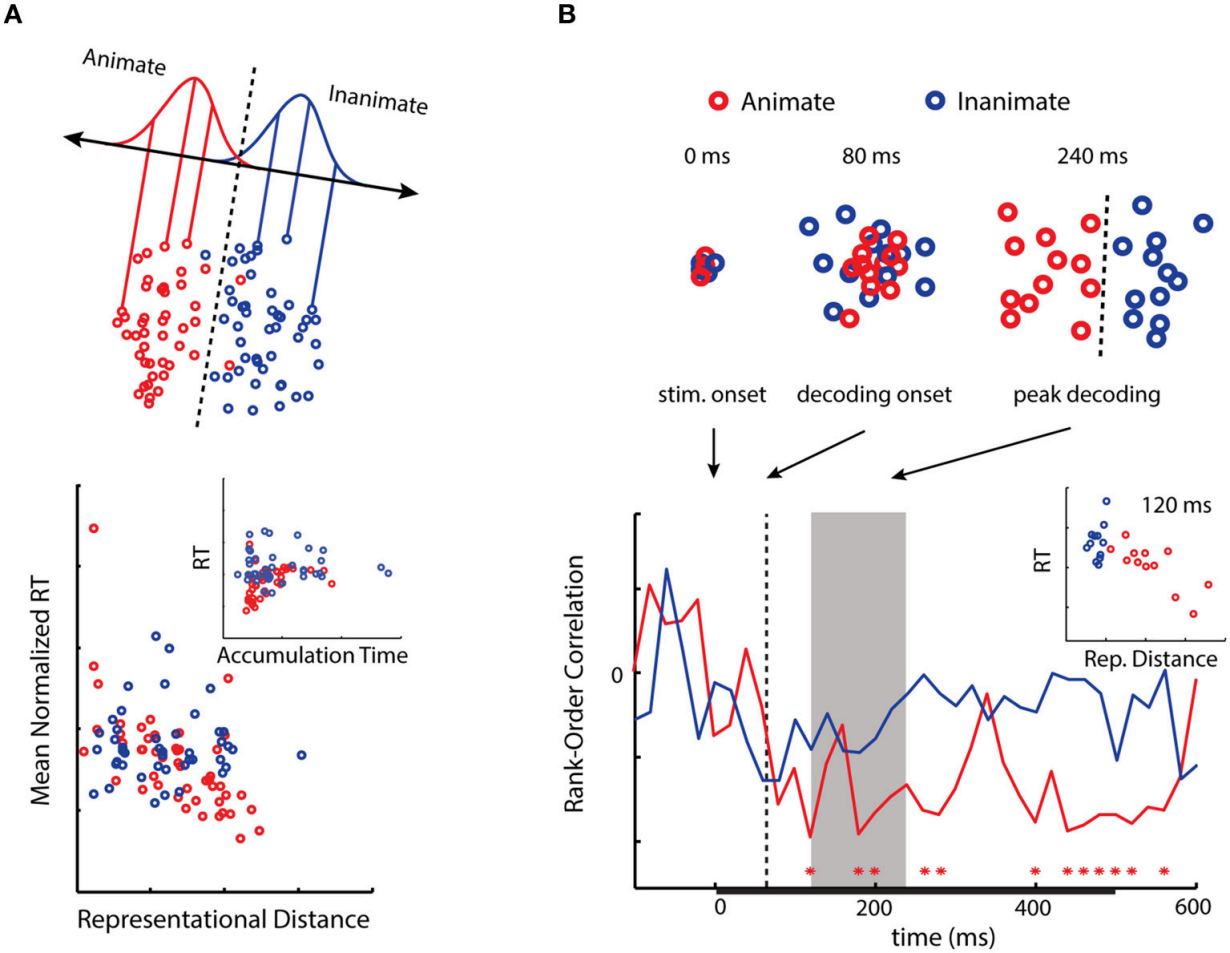
**(A)** A 2D activation space for animacy in human IT. Distances from a decision boundary for animacy in human IT negatively correlate with RT as predicted from the neural distance-to-bound approach. When distance is transformed into a drift rate parameter, RT and evidence accumulation time positively correlate (modified from Carlson et al., 2014; © 2014 by the Massachusetts Institute of Technology). **(B)** Emergent activation spaces for animacy as reconstructed using MEG. Distances from decision boundaries at peak decoding (gray area) negatively correlate with RT (modified from Ritchie et al., 2015). Interestingly, for both results the correlations are primarily driven by the animate exemplar stimuli (for discussion, see Carlson et al., 2014).

Inferior temporal cortex (IT) has been strongly implicated in object categorization in humans and primates (Logothetis and Sheinberg, 1996), and information about object categories—in particular, animacy—is highly decodable from this region using fMRI (Kriegeskorte et al., 2008b; Connolly et al., 2012; Konkle and Caramazza, 2013). In our first experiment we asked whether the animacy information latent in activity patterns in this region might be utilized when subjects performed an object categorization task (Carlson et al., 2014). Using the fMRI data of Kriegeskorte et al. (2008b), we computed the representational distances of activity patterns for 92 object exemplars (faces and bodies of humans and animals, as well as natural objects and human artifacts) from a decision boundary for animacy in IT. We then correlated these distances with the mean RT of separate subjects performing the animacy task. Despite using neural and behavioral data from completely different subjects, we observed a significant negative correlation between RT and the representational distances (Figure 2A). This result suggests that animacy information in activity patterns in the region may also be used by the observers performing the animacy task.

While typically utilized with fMRI, MVPA is also increasingly being employed with EEG/MEG (King and Dehaene, 2014). It has been shown that significant decoding for object categories, and in particular animacy, occurs as early as 60 ms post-stimulus onset, with peak classifier performance occurring at greater latencies for more abstract categories (Carlson et al., 2013; Cichy et al., 2014). In our second experiment we sought to determine when in time we might observe a negative correlation between RT and distance-to-bound (Ritchie et al., 2015). Peak decoding reflects the time at which information about stimulus categories is most discriminable in the brain, thus we predicted representational distance would negatively correlate with RTs during the period of peak decoding. We estimated the representational distances at each 20 ms time-point -100–600 ms post-stimulus onset for 24 object exemplars (same subordinate groupings as in our previous experiment). While in the MEG, subjects performed the animacy task, and their median RTs were correlated with the representational distances at each time point. This allowed us to see when in time there was a significant correlation between representational distance and RT. As predicted, we observed a significant correlation during the period of peak decoding, as well as at later time points (Figure 2B). More generally we found that the relationship between representational distance and RT followed the time-course of decoding.

Taken together, these two results provide compelling evidence in support of the viability of the neural distance-to-bound approach.

### 3.2. Distance-to-bound and the neural basis of decision-making

The neural distance-to-bound approach has two important implications for research on the neural basis of decision-making. First, it has been suggested, in part based on decoding methodology, that the line between representing and deciding in the brain is blurred (DiCarlo and Cox, 2007). Our approach provides theoretical and empirical support for this perspective. If an observer's decision boundary extends through an activation space, then at least in some circumstances stimulus representations and decision variables may be implemented in the same neural component (Carlson et al., 2014). This contrasts with perspectives according to which stimulus representations and decision variables are generally associated with distinct brain regions (Schall, 2001; Heekeren et al., 2004; Shadlen et al., 2007; Filimon et al., 2013).

Second, evidence accumulation models of choice and RT are a popular means of investigating the neural underpinnings of decision-making (Smith and Ratcliff, 2004; Gold and Shadlen, 2007; Shadlen and Kiani, 2013). While there are several versions of these models to choose from Ratcliff and Smith (2004) they all share certain features: evidence is sampled from a random variable; at each iteration of the model, the evidence is used to update a decision variable; and when the decision variable reaches a stopping value, or threshold, the observer makes a decision. Typically, choice and RT effects are modeled as resulting from differences in accumulation rate of the decision variable between experimental conditions.

Distance-to-bound and evidence accumulation models of RT have sometimes been contrasted with each other (Pike, 1973; Thomas, 2006). However, this opposition is not obligatory, since distance-to-bound can be related to accumulation rate (Ratcliff, 1985; Ashby, 2000). We assume the simplest RT-distance relationship possible: a monotonic decrease in RT correlating with a monotonic increase in distance. So neural distance-to-bound can also be thought of as an approach for characterizing the distance between the accumulate rate distributions (Figures 1D–E), for any evidence accumulation model that assumes a monotonic decrease in RTs as accumulation rate increases. Thus, neural distance-to-bound also provides a method for connecting neural decoding and neural evidence accumulation approaches.

To illustrate the connection, we used distances from the animacy boundary in human IT to simulate accumulation rates for the sequential probability ratio test (SPRT; Wald, 1945), which has been used to relate spike rates to evidence accumulation (Gold and Shadlen, 2002). SPRT is a dynamic extension of classic SDT: the observer selects a response based on the log-likelihood ratio, but if the value of the ratio has not yet reached threshold, the observer receives another unit of evidence. The decision variable is the running tally of the ratio, which accumulates until the threshold is reached. In our study, we simulated SPRT using the representational distances in human IT for each individual exemplar, transforming the distances into accumulation rates (Figure 2A; Carlson et al., 2014).

## 4. The path to an inner psychophysics: still a long way to go

We believe neural distance-to-bound has considerable potential as an (easy to apply) approach for developing representational linking hypotheses. Still, we stress that it is just one possible approach for furthering the study of inner psychophysics. In some domains of perception and cognition, it might not be viable at all. For example, it is unclear how well it will fair in a domain like neurolinguistics, where there is considerable difficulty in linking the primitives of linguistic theory to the brain (Poeppel, 2012). Furthermore, other approaches that may be superior at modeling RT, such as exemplar models (Nosofsky and Stanton, 2005), could provide a better connection between activation spaces and evidence accumulation.

More fundamentally, failure to observe a negative RT-distance correlation does not necessarily entail that the information in neural activity is unused. Instead, we might have the wrong model for *how* it is used. For instance, crucially our approach assumes linear separability, as using nonlinear classifiers for decoding is typically discouraged on the grounds that they are overpowered and lack biological plausibility (Kamitani and Tong, 2005; DiCarlo and Cox, 2007). However, many quantitative models of categorization do not share this assumption. For example, exemplar models have often been tested using stimulus sets that do not allow for linear separation in perceptual space (e.g., Nosofsky, 1986). Thus, the existence of psychologically plausible nonlinear categorization models may warrant revisiting the use of nonlinear classifiers in MVPA.

The ultimate import of our approach, then, is that it suggests more sophisticated representational linking hypotheses are possible. Recognizing this possibility is an important step along the path to an inner psychophysics, and the realization of Fechner's dream.

## Author contributions

JR and TC contributed equally to developing the ideas presented in the paper. JR wrote, and TC helped edit, the manuscript.

## Funding

This research was supported by an Australian Research Council Future Fellowship [FT120100816] to TC. The funders had no role in the preparation or decision to publish the manuscript.

### Conflict of interest statement

The authors declare that the research was conducted in the absence of any commercial or financial relationships that could be construed as a potential conflict of interest.
